# Supplementary material for: Bone Morphogenetic Proteins for Nucleus Pulposus Regeneration
Source: Int J Mol Sci. 2020 Apr 14;21(8):2720. doi: 10.3390/ijms21082720 (PMC7215319; doi:10.3390/ijms21082720)
Supplement: Supplementary file 1 [file ijms-21-02720-s001.pdf]

Supplementary data

1.1.1. The effect of TGF-β on matrix production by NP cells

NP cells of 2 donors were seeded in high density on 0.12 cm^2 type II collagen-coated polycarbonate film (PCF) 0.4 µm transwell filters (Merck Millipore, Billerica, MA, USA) at passage 2 at $1*10^6$ cells/cm^2 with differentiation culture medium (DMEM (Dulbecco's Modified Eagle Medium, low glucose, pyruvate, L-glutamine, 31885, Gibco, Carlsbad, CA, USA) + 2% insulin-transferrin-selenium-X (Gibco) + 0.4 nM ASAP (Sigma, St. Louis, MO, USA) + 2% human serum albumin (Sanquin, Amsterdam, The Netherlands) + 100 U/ml penicillin + 100 µg/ml streptomycin (pen/strep, Gibco). TGF-β1 or TGF-β2 (R&D Systems, Minneapolis, MA, USA) were added to the culture medium at 4 nM and 0.4 nM (100 and 10 ng/ml, respectively) and compared to differentiation medium without addition of a growth factor (control). Cultures lasted 28 days.

NP cells are known for their limited matrix production and need to be stimulated by growth factors. Here, we looked at the difference between TGF-β1, TGF-β2, and no TGF-β added to differentiation medium. Figure S1 shows no differences in GAG, DNA or GAG/DNA between both types of TGF-β or the concentration at which they were used, but there was notably less proliferation or matrix production without added growth factor.

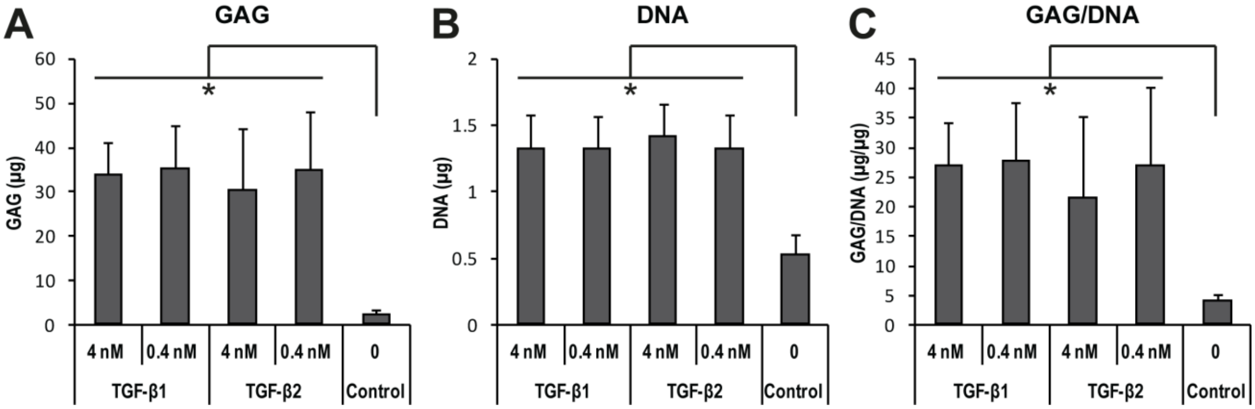

Figure S1 – Human degenerated nucleus pulposus cells were cultured on transwells coated with type II collagen in differentiation culture medium with or without the addition of TGF-β. (**A**) GAG content. (**B**) DNA content. (**C**) GAG corrected for DNA. Significant differences are noted as follows: * p≤0.001.
